# Effect of Noradrenaline on the Facial Stimulation-Evoked Mossy Fiber-Granule Cell Synaptic Transmission in Mouse Cerebellar Cortex

**DOI:** 10.3389/fnins.2021.785995

**Published:** 2021-11-15

**Authors:** Bing-Xue Li, Hua Jin, Guang-Jian Zhang, Li-Na Cui, Chun-Ping Chu, De-Lai Qiu

**Affiliations:** ^1^Brain Science Research Center, Yanbian University, Yanji, China; ^2^Department of Physiology and Pathophysiology, College of Medicine, Yanbian University, Yanji, China; ^3^Department of Psychology, Affiliated Hospital of Yanbian University, Yanji, China; ^4^Department of Pain, Affiliated Hospital of Yanbian University, Yanji, China; ^5^Department of Acupuncture, Affiliated Hospital of Yanbian University, Yanji, China

**Keywords:** adrenergic receptor (AR), cerebellum, facial stimulation, electrophysiology, mossy fiber-granule cell synaptic transmission

## Abstract

Noradrenaline is an important neuromodulator in the cerebellum. We previously found that noradrenaline depressed cerebellar Purkinje cell activity and climbing fiber–Purkinje cell synaptic transmission *in vivo* in mice. In this study, we investigated the effect of noradrenaline on the facial stimulation-evoked cerebellar cortical mossy fiber–granule cell synaptic transmission in urethane-anesthetized mice. In the presence of a γ-aminobutyrate_A_ (GABA_A_) receptor antagonist, air-puff stimulation of the ipsilateral whisker pad evoked mossy fiber–granule cell synaptic transmission in the cerebellar granular layer, which expressed stimulus onset response, N1 and stimulus offset response, N2. Cerebellar surface perfusion of 25 μM noradrenaline induced decreases in the amplitude and area under the curve of N1 and N2, accompanied by an increase in the N2/N1 ratio. In the presence of a GABA_A_ receptor blocker, noradrenaline induced a concentration-dependent decrease in the amplitude of N1, with a half-maximal inhibitory concentration of 25.45 μM. The noradrenaline-induced depression of the facial stimulation-evoked mossy fiber–granule cell synaptic transmission was reversed by additional application of an alpha-adrenergic receptor antagonist or an alpha-2 adrenergic receptor antagonist, but not by a beta-adrenergic receptor antagonist or an alpha-1 adrenergic receptor antagonist. Moreover, application of an alpha-2 adrenergic receptor agonist, UK14304, significantly decreased the synaptic response and prevented the noradrenaline-induced depression. Our results indicate that noradrenaline depresses facial stimulation-evoked mossy fiber–granule cell synaptic transmission via the alpha-2 adrenergic receptor *in vivo* in mice, suggesting that noradrenaline regulates sensory information integration and synaptic transmission in the cerebellar cortical granular layer.

## Introduction

The cerebellar cortex acquires information from three classes of afferents: mossy fibers (MFs), climbing fibers, and multilayered fibers, and generates motor-related output by Purkinje cells (PCs) (Haines and Dietrichs, 2002). Under *in vivo* conditions, granule cells (GCs) exhibit a low frequency of spontaneous firing, but they are very sensitive to sensory stimulation (van Beugen et al., 2013). This sensory stimulation induces spike firing followed by a GABAergic inhibitory response in the GCs (Eccles et al., 1967; Ito, 1984; Jakab and Hámori, 1988), which precisely encodes the sensory information (D’Angelo et al., 2005; Jörntell and Ekerot, 2006). Therefore, it has been suggested that the GCs both exhibit high-frequency and high-fidelity properties in response to sensory stimulation, which could ensure that accurate information is transmitted to PCs (Arenz et al., 2008; van Beugen et al., 2013; Bing et al., 2015), while also filtering out unassociated components (Chadderton et al., 2004).

Noradrenaline (NA) is a widely studied neuromodulator involved in the modulation of learning and memory in the central nervous system. Anatomical studies indicate that noradrenergic (NAergic) fibers originate in the locus coeruleus (LC) and distribute through the cerebellar cortex through a multilayered fiber pathway (Kimoto et al., 1978; Schroeter et al., 2000). Noradrenergic inputs of the cerebellum have been shown to be involved in cerebellum-dependent motor learning (McCormick and Thompson, 1982; Keller and Smith, 1983; Watson and McElligott, 1984; Pompeiano, 1998) and long-term depression induction at PF–PC synapses in the flocculus by activating protein kinase A (PKA) (Inoshita and Hirano, 2021). Either iontophoretic application of NA or activation of the LC-induced potentiation of GABAergic transmission at molecular layer interneurons–PC synapse results in an inhibition of the PC spontaneous simple spike activity via activation of adrenoceptors (ARs) (Mitoma and Konishi, 1999; Saitow et al., 2000).

The ARs are G-protein-coupled receptors that come in two types, α-AR and β-AR. Both α-ARs and β-ARs are present in the cerebellar cortex, including the granular layer (GL) (McCune et al., 1993). The roles of α-ARs and β-ARs in the cerebellar cortex vary. Several studies demonstrated that NA could regulate cerebellar-dependent learning tasks and long-term memory via activation of β-ARs (Cartford et al., 2004; Schambra et al., 2005; Hein, 2006). *In vitro*, NA facilitated mouse cerebellar parallel fiber–PC synaptic transmission via activation of β-ARs, but it suppressed synaptic transmission via α2-ARs (Lippiello et al., 2015). However, NA facilitated spontaneous inhibitory postsynaptic currents of PCs via simultaneous activation of both α1-ARs and β-ARs located at the presynaptic terminals of molecular layer interneurons, which could synergically boost GABAergic transmitter release (Hirono et al., 2014). In addition, activation of α2-ARs by NA decreased the probability of transmitter release at climbing fiber–PC synapses, which in turn suppressed the climbing fiber-evoked dendritic calcium transients and controlled the induction of synaptic plasticity at parallel fiber–PC synapses by modulating dendritic calcium influx (Carey and Regehr, 2009). We previously found that NA-activated presynaptic α2-AR regulated climbing fiber–PC synaptic transmission via the PKA signaling pathway, suggesting that the NAergic fibers from the nucleus of the LC might regulate the output behavior of PC by suppressing the information transmission from the inferior olivary nucleus to the cerebellar cortex *in vivo* in mice (Sun et al., 2019; Cui et al., 2020).

Taken together, the effects of NA on cerebellar cortical neuronal synaptic transmission have been well studied in vitro, but the modulatory function of NA on sensory information processing in the cerebellar GL is not well understood. Therefore, in this study, we combined electrophysiological and pharmacological approaches to investigate the effects of NA on the facial stimulation-evoked MF-GC synaptic transmission in the absence the GABAergic inhibition in urethane-anesthetized mice.

## Materials and Methods

All the experimental procedures were approved by the Animal Care and Use Committee of Yanbian University and performed in accordance with the animal welfare guide lines of the National Institutes of Health. The permit number is SYXK (Ji) 2011-006. Anesthesia and surgical procedures have been described previously (Chu et al., 2011). In brief, either male (*n* = 36) or female (*n* = 30) adult (6–8 weeks old) ICR mice were anesthetized with urethane (1.1 g/kg body weight, intraperitoneal injection, i.p). After a water tight chamber was prepared, a 1–1.5 mm craniotomy was opened to expose the cerebellar surface of Crus II. The brain surface was superfused with oxygenated artificial cerebrospinal fluid (ACSF: 125 mM NaCl, 3 mM KCl, 1 mM MgSO_4_, 2 mM CaCl_2_, 1 mM NaH_2_PO_4_, 25 mM NaHCO_3_, and 10 mM D-glucose) with a peristaltic pump (Gilson Minipulse 3; Villiers, LeBel, France). The rectal temperature was monitored, and keeped at 37.0 ± 0.2°C.

The sensory stimulation was performed by air-puff (60 ms, 50–60 psi) of the ipsilateral whisker pad through a 12-gauge stainless steel tube connected to a pressurized injection system (Picospritzer^®^ III; Parker Hannifin Co., Pine Brook, Fairfield, NJ, United States). The whiskers were cut off to avoid the stimulation of the whiskers. The air-puff stimuli were controlled by a personal computer and were synchronized with the electrophysiological recordings and delivered at 0.05 Hz via a Master 8 controller (A.M.P.I., Jerusalem, Israel) and Clampex 10.4 software.

**FIGURE 1:**
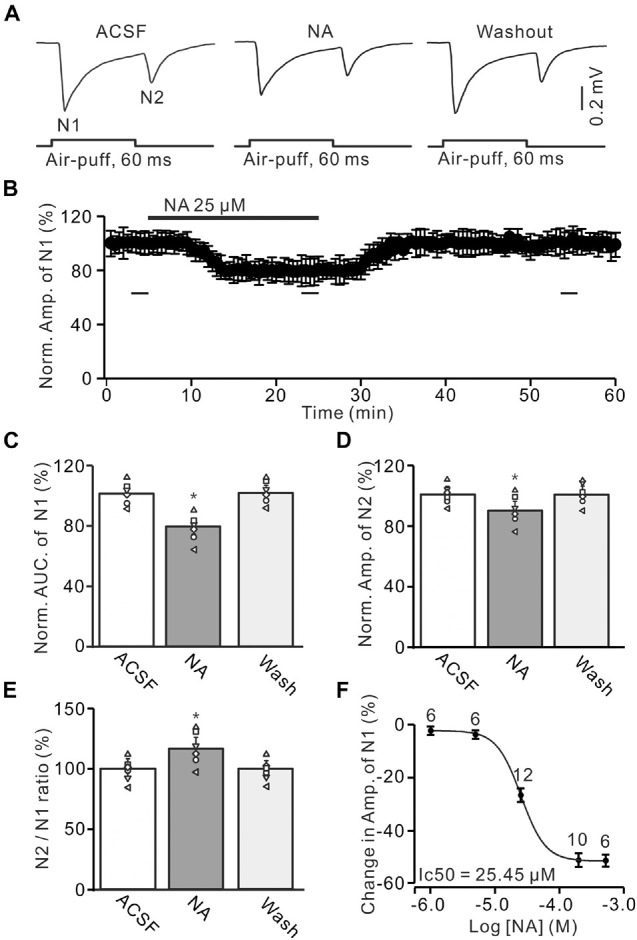
Noradrenaline (NA) depresses the facial stimulation-evoked mossy fiber-granule cell (MF-GC) synaptic transmission in mouse cerebellar cortex. **(A)** Representative field potential recording traces showing the air-puff stimulation (60 ms, 60 psi) evoked MF-GC synaptic transmission in a mouse cerebellar GL during treatment with artificial cerebrospinal fluid (ACSF), NA (25 μM) and recovery (washout). **(B)** Summary of data showing the time course of normalized amplitude of N1 during NA application. Bars denote the data points which were used for panels **(C–E)** in treatments with ACSF, NA, and washout. **(C)** Mean value (± SEM) with individual data showing the normalized area under the curve (AUC) of N1 in treatments with ACSF, NA, and recovery (washout). **(D,E)** Mean value (± SEM) with individual data showing the normalized amplitude of N2 (D) and the N2/N1 ratio (E) in treatments with ACSF, NA and recovery (washout). **(F)** The concentration-response curve shows the NA-induced decrease in amplitude of the facial stimulation-evoked of N1. The IC_50_ value obtained from the curve was 25.45 μM. The number of recordings tested for each concentration is indicated near the bars. *p < 0.05 versus control (ACSF); *n* = 6 in each group.

Local field potential recordings from GL were performed with an Axopatch 200B amplifier (Molecular Devices, Foster City, CA, United States) under current clamp conditions (*I* = 0). The potentials were acquired through a Digidata 1440 series analog-to-digital interface on a personal computer using Clampex 10.4 software. Recording electrodes were filled with ACSF and with resistances of 3–5 MΩ. Air-puff (60 ms, 50–60 psi) of the ipsilateral whisker pad evoked a paired-negative components N1, N2, accompanied with a positive component P1 in the GL of cerebellar cortical folium Crus II (Figure 1A). According to our previous studies (Wu et al., 2014; Bing et al., 2015; Ma et al., 2019), N1 and N2 were identified as MF-GC synaptic transmission which evoked by the stimulation-on (N1) and stimulation-off (N2), respectively. P1 was identified as GABAergic inhibitory components which could be abolished by GABA_A_ receptor blocker.

The reagents included urethane; NA; phentolamine (Phen), nonselective α-AR antagonist; propranolol (Prop), a nonselective β-AR blocker; prazosin (Praz), α1-AR antagonist; yohimbine (Yohim), α2-AR antagonist; UK14304 (UK), α2-AR agonist and gabazine (SR95531) were bought from Sigma-Aldrich (Shanghai, China). The chemicals were dissolved in ACSF and applied to the cerebellar surface at 0.5 ml/min by a peristaltic pump (Gilson Minipulse 3; Villiers, Le Bel, France). The ACSF included gabazine (20 μM) during all recordings to prevent GABA_A_ receptor-mediated inhibition.

Electrophysiological data were analyzed using Clampfit 10.4 software (Molecular Device, Foster City, CA, United States). The amplitude and area under the curve (AUC) of the evoked field potential responses were maintained constant for an individual experiment in treatments of ACSF, drugs and recovery. It has been suggested that changes in the N2/N1 ratio vary inversely with the presynaptic release of transmitter (Mennerick and Zorumski, 1995; Hashimoto and Kano, 1998). We calculated N2/N1 ratio to mirror the probability of vesicular release at the MF-GC synapse (Zhang et al., 2020). All data are expressed as the mean ± SEM. Differences between the mean values recorded under control and test conditions were evaluated with the one-way ANOVA with Tukey’s post-hoc test using SPSS (Chicago, IL, United States) software. *P* values below 0.05 were considered to indicate a statistically significant difference between experimental groups.

## Results

### Noradrenaline Depressed Facial Stimulation-Evoked Mossy Fiber-Granule Cell Synaptic Transmission in Granular Layer via α-Adrenoceptors

Air-puff stimulation on the ipsilateral whisker pad evoked field potential responses in the GL (depth: 300 μm), which expressed strong negative components N1 and N2, accompanied with a positive component P1 in the GL of the cerebellar cortical folium Crus II (Figure 1A). Based on our previous studies (Wu et al., 2014; Bing et al., 2015; Ma et al., 2019), N1 and N2 were identified as MF-GC synaptic transmission, while P1 was the GABAergic inhibitory component (Ma et al., 2019). To study the effect of NA on MF-GC synaptic transmission, we recorded the facial stimulation-evoked field potential response in the GL in the absence of GABAergic inhibition. In the presence of the GABA_A_ receptor blocker, gabazine (20 μM), air-puff stimulation (60 ms, 60 psi) of the ipsilateral whisker pad induced N1 and N2 in the GL (Figures 1A,B). Cerebellar surface perfusion of NA (25 μM) decreased the amplitude and area under the curve (AUC) of N1 (Figure 1A). In the presence of NA, the normalized amplitude of N1 was 79.1 ± 5.5% of baseline [ACSF: 100.1 ± 4.9%; *F* (3, 54) = 13.25, *P* = 0.023; *n* = 6; not shown], and the normalized AUC of N1 was 79.5 ± 5.6% of baseline [99.8 ± 4.6%; *F* (3, 60) = 14.76, *P* = 0.029; *n* = 6; Figure 1C]. In addition, the application of NA decreased the normalized amplitude of N2 to 90.4 ± 5.8% of baseline [ACSF: 100.1 ± 4.3%; *F* (2, 28) = 10.92, *P* = 0.041; *n* = 6; Figure 1D]. However, NA produced a significant increase in the N2/N1 ratio from baseline (ACSF: 99.7 ± 4.9%) to 114.3 ± 6.3% [*F* (2, 33) = 14.33, *P* = 0.021; *n* = 6; Figure 1E]. The NA-produced inhibition of the amplitude of N1 was concentration-dependent. The lowest effective dose was 5 μM, which decreased the amplitude of N1 to 94.95 ± 5.3% of baseline [ACSF: 100.1 ± 4.4%; *F* (2, 39) = 12.94, *P* = 0.031; *n* = 6], while the maximum effective dose was 500 μM, which decreased the amplitude of N1 to 52.3 ± 5.9% of baseline [ACSF: 99.9 ± 4.6%; *F* (3, 63) = 17.57, *P* = 0.013; *n* = 10; Figure 1F]. The half-maximal inhibitory concentration (IC_50_) of NA was 25.45 μM. These results indicate that NA depresses the facial stimulation-evoked MF-GC synaptic transmission in a concentration-dependent manner.

**FIGURE 2:**
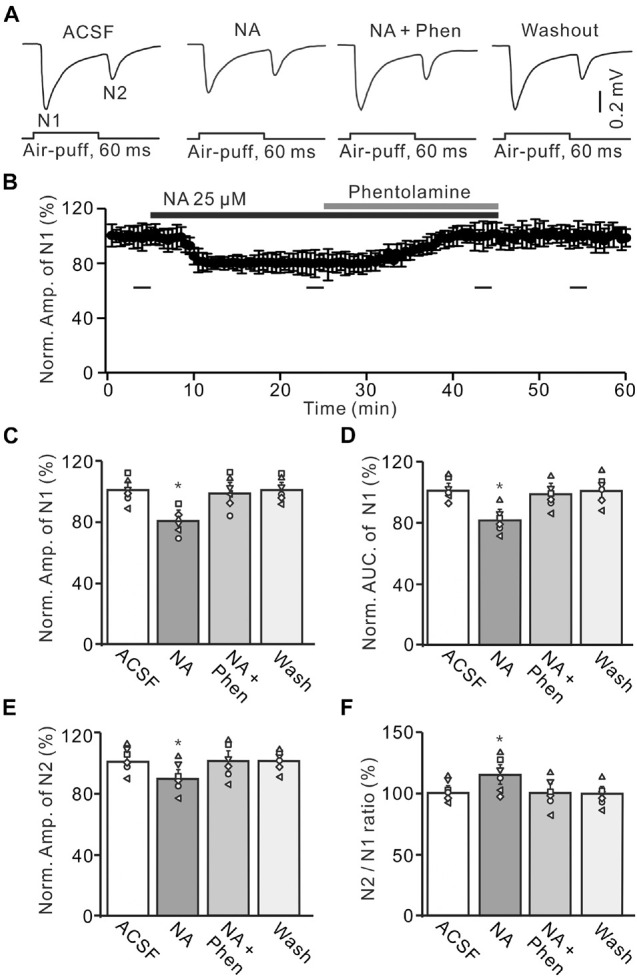
Application of α-adrenoceptor (AR) antagonist reverses the NA-induced depression of the facial stimulation-evoked MF-GC synaptic transmission. **(A)** Representative field potential traces showing the facial stimulation (60 ms, 60 psi) evoked MF-GC synaptic transmission in a mouse cerebellar GL during treatment with ACSF, NA (25 μM), NA + phentolamine (Phen; 100 μM), and recovery (washout). **(B)** Summary of data showing the time course of normalized amplitude of N1 during treatment with ACSF, NA (25 μM), NA + phentolamine (Phen; 100 μM) and recovery (washout). Bars denote the data points which were used in the bar graphs **(C–F)** in treatments with ACSF, NA, NA + phentolamine and washout. **(C,D)** Mean value (± SEM) with individual data showing the normalized amplitude **(C)** and AUC **(D)** of N1 during each treatment, NA, NA + Phen and recovery (washout). **(E,F)** Mean value (± SEM) with individual data showing the normalized amplitude of N2 **(E)** and the N2/N1 ratio **(F)** during each treatment. Note that application of phentolamine reversed the NA induced-inhibition of facial stimulation-evoked MF-GC synaptic transmission. *p < 0.05 versus control (ACSF); *n* = 6 in each group.

We further employed a nonselective α-AR antagonist, phentolamine (Phen), to determine whether NA induced inhibition of MF-GC synaptic transmission through α-ARs. Application of NA produced a significant decrease in the amplitude of N1, which was completely reversed by additional application of Phen (100 μM) (Figures 2A,B). In the presence of a mixture of Phen and NA, the normalized amplitude of N1 increased from 79.3 ± 5.1% (NA, 25 μM) to 100.2 ± 5.9% [*F* (2, 19) = 11.76, *P* = 0.045; *n* = 6] of baseline [ACSF: 100.1 ± 5.0%, *F* (1, 5) = 0.021, *P* = 0.89; *n* = 6; Figure 2C], and the normalized AUC of N1 increased from 80.1 ± 5.1% (NA) to 98.6 ± 5.3% [*F* (3, 54) = 14.82, *P* = 0.021; *n* = 6] of baseline [ACSF: 99.7 ± 4.3 %, *F* (1, 5) = 0.005, *P* = 0.95; *n* = 6; Figure 2D]. Additional perfusion of Phen also reversed the NA-induced inhibition of N2. The normalized amplitude of N2 increased from 89.8 ± 5.8% (NA, 25 μM) to 100.3 ± 6.1% [NA + Phen; *F* (3, 39) = 13.64, *P* = 0.024; *n* = 6] of baseline [ACSF: 100.2 ± 5.0%; *F* (1, 7) = 0.34, *P* = 0.59; *n* = 6; Figure 2E]. Moreover, the N2/N1 ratio decreased from 113.2 ± 6.1% (NA, 25 μM) to 100.1 ± 5.9% [NA + Phen; *F* (3, 60) = 17.24, *P* = 0.015; *n* = 6] of baseline [ACSF: 100 ± 4.1%; *F* (1, 5) = 0.003, *P* = 0.96; *n* = 6; Figure 2F]. These results indicate that application of an α-AR antagonist reverses the NA-induced inhibition of facial stimulation-evoked MF-GC synaptic transmission.

**FIGURE 3:**
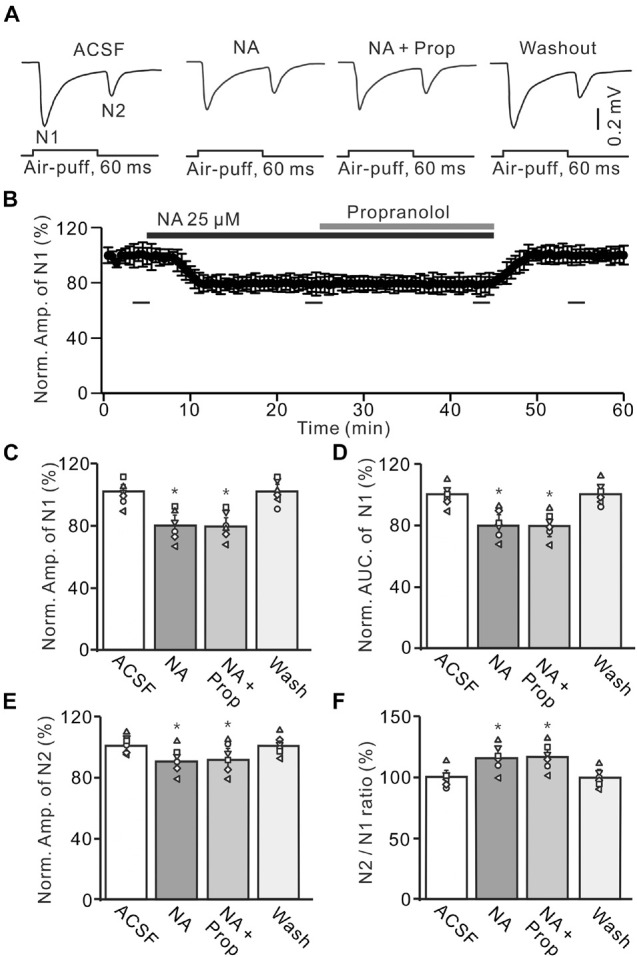
Application of β-AR antagonist does less affect the NA-induced inhibition of the facial stimulation-evoked MF-GC synaptic transmission. **(A)** Representative field potential traces showing the facial stimulation (60 ms, 60 psi) evoked MF-GC synaptic transmission in a mouse cerebellar GL during treatment with ACSF, NA (25 μM), NA + propranolol (Prop; 100 μM), and recovery (washout). **(B)** Mean value (± SEM) with individual data showing the time course of normalized amplitude of N1 during treatment with ACSF, NA, NA + propranolol (Prop; 100 μM) and recovery (washout). Bars denote the data points which were used in the bar graphs **(C–F)** in treatments with ACSF, NA, NA + propranolol and washout. **(C,D)** Mean value (± SEM) with individual data showing showing the normalized amplitude **(C)** and AUC **(D)** of N1 during each treatment. **(E,F)** Mean value (± SEM) with individual data showing the normalized amplitude of N2 **(E)** and the N2/N1 ratio **(F)** for each treatment. Note that propranolol failed to affect the NA induced inhibition of facial stimulation-evoked MF-GC synaptic transmission. *p < 0.05 versus control (ACSF); *n* = 6 in each group.

We also employed a nonselective β-ARs antagonist, propranolol (Prop, 100 μM) to determine whether NA induced inhibition of MF-GC synaptic transmission through β-ARs. Additional application of 100 μM Prop failed to reverse the NA-induced inhibition of N1 (Figures 3A,B). In the presence of a mixture of Prop and NA, the amplitude of N1 was 78.7 ± 5.5% (NA + Prop; *n* = 6) of baseline [ACSF: 99.9 ± 4.9%; *F* (2, 39) = 12.61, *P* = 0.036; *n* = 6], which was similar to that in the presence of NA alone [NA: 79.3 ± 5.5%; *F* (1, 7) = 1.52, *P* = 0.33; *n* = 6; Figure 3C], and the normalized AUC of N1 was 80.5 ± 5.3% (NA + Prop; *n* = 6) of baseline [ACSF: 99.8 ± 4.7%; *F* (3, 60) = 17.92, *P* = 0.012; *n* = 6], which was not significantly different from that observed in the presence of NA alone [NA: 79.2 ± 5.4%; *F* (1, 4) = 0.009, *P* = 0.16; *n* = 6; Figure 3D]. Additional perfusion of Prop did not reverse the NA-induced inhibition of N2. In the presence of Prop and NA, the normalized amplitude of N2 was 89.8 ± 5.6% (NA + Prop; *n* = 6) of baseline [ACSF: 100.1 ± 3.6%; *F* (3, 60) = 15.03, *P* = 0.018; *n* = 6], which was not significantly different from that observed in the presence of NA alone [90.3 ± 5.3%; *F* (1, 7) = 0.02, *P* = 0.74; *n* = 6; Figure 3E]. Moreover, the N2/N1 ratio increased to 114.1 ± 5.3% (NA + Prop; *n* = 6) of baseline [ACSF: 100 ± 4.1%; *F* (3, 54) = 14.86, *P* = 0.014; *n* = 6], which was similar to that observed in the presence of NA alone [NA: 113.9 ± 5.4%; *F* (1, 5) = 0.32, *P* = 0.67; *n* = 6; Figure 3F]. These results indicate that blockade of β-AR does not block the NA-induced inhibition of facial stimulation-evoked MF-GC synaptic transmission.

### Noradrenaline Depressed Cerebellar Mossy Fiber–Granule Cell Synaptic Transmission Through α2-Adrenoceptor *in vivo* in Mice

**FIGURE 4:**
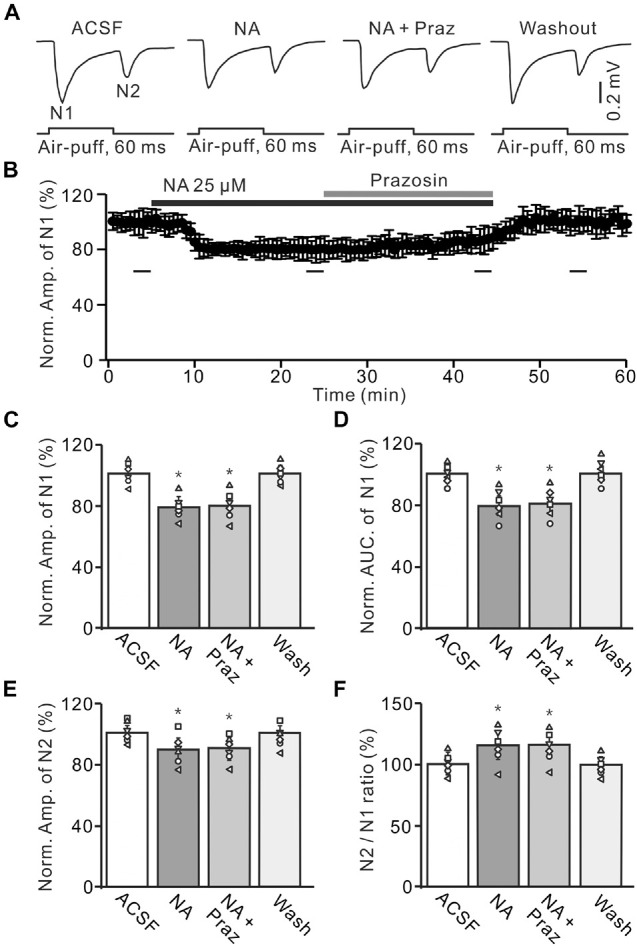
Application of α1-AR blocker, prazosin failes to reverse the NA-induced depression of the facial stimulation-evoked MF-GC synaptic transmission. **(A)** Representative field potential traces showing the facial stimulation (60 ms, 60 psi) evoked MF-GC synaptic transmission in a mouse cerebellar GL during treatment with ACSF, NA (25 μM), NA + prazosin (Praz; 50 μM), and recovery (washout). **(B)** Summary of data showing the time course of normalized amplitude of N1 during treatment with ACSF, NA, NA + prazosin (Praz; 50 μM) and recovery (washout). Bars denote the data points which were used in the bar graphs **(C–F)** in treatments with ACSF, NA, NA + prazosin and washout. **(C,D)** Mean value (± SEM) with individual data showing the normalized amplitude **(C)** and AUC **(D)** of N1 during each treatment. **(E,F)** Mean value (± SEM) with individual data showing the normalized amplitude of N2 **(E)** and the N2/N1 ratio **(F)** for each treatment. *p < 0.05 versus control (ACSF); *n* = 6 in each group.

A previous study has shown that both α1-ARs and α2-ARs are expressed in the cerebellar GL (Schambra et al., 2005). We then examined the effects of the α1-AR antagonist, prazosin (Praz), on the NA-induced depression of MF-GC synaptic transmission. Application of NA produced a significant decrease in amplitude of N1, which was not reversed by additional application of Praz (50 μM) (Figures 4A,B). In the presence of a mixture of Praz (50 μM) and NA, the amplitude of N1 was 78.2 ± 5.7% (NA + Praz; *n* = 6) of baseline [ACSF: 100 ± 4.3%; *F* (2, 33) = 11.63, *P* = 0.042; *n* = 6], which was similar to that observed in the presence of NA alone [NA: 78.7 ± 5.0%; *F* (1, 5) = 0.01, *P* = 0.58; *n* = 6; Figure 4C], and the normalized AUC of N1 was 81.1 ± 5.7% of baseline [ACSF: 100.4 ± 4.0%; *F* (3, 54) = 13.92, *P* = 0.023; *n* = 6], which was not significantly different from that observed in the presence of NA alone [NA: 80.3 ± 5.7%; *F* (1, 5) = 0.24, *P* = 0.41; *n* = 6; Figure 4D]. Additional perfusion of Praz did not reverse the NA-induced inhibition of N2. In the presence of a mixture of Praz and NA, the normalized amplitude of N2 was 90.2 ± 5.0% of baseline [ACSF: 99.9 ± 3.9%; *F* (3, 60) = 17.67, *P* = 0.015; *n* = 6], which was not significantly different from that observed in the presence of NA alone [NA: 89.4 ± 5.9%; *F* (1, 5) = 0.27, *P* = 0.32; *n* = 6; Figure 4E]. Moreover, the N2/N1 ratio increased to 115.3 ± 6.2% of baseline [ACSF: 99.8 ± 4.4%; *F* (3, 60) = 17.32, *P* = 0.016; *n* = 6], which was similar to that observed in the presence of NA alone [NA: 115 ± 6.7%; *F* (1, 5) = 0.36, *P* = 0.54; *n* = 6; Figure 4F]. These results indicate that blocking α1-AR does not reverse the NA-induced depression of the facial stimulation-evoked MF-GC synaptic transmission in the mouse cerebellar cortex.

**FIGURE 5:**
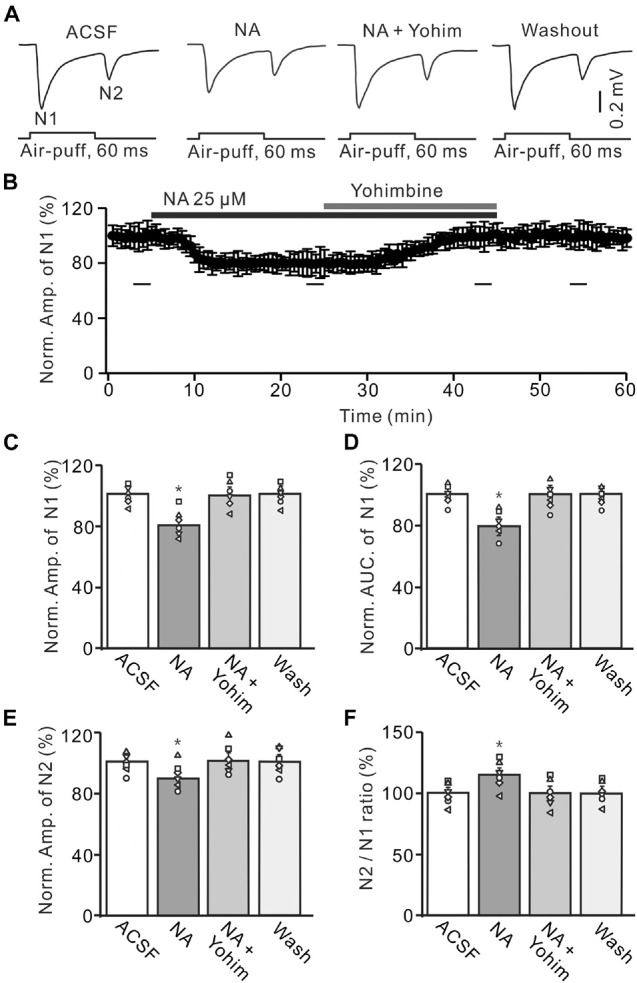
Application of yohimbine reverses the NA-induced depression of the MF-GC synaptic transmission. **(A)** Representative field potential traces showing the facial stimulation (60 ms, 60 psi) evoked responses in a mouse cerebellar GL during treatment with ACSF, NA (25 μM), NA + yohimbine (Yohim; 100 μM), and recovery (washout). **(B)** Summary of data (*n* = 6) showing the time course of normalized amplitude of N1 during treatment with ACSF, NA, NA + yohimbine (Yohim; 100 μM) and recovery (washout). **(C,D)** Bar graphs with individual data showing show the normalized amplitude **(C)** and AUC **(D)** of N1 during each treatment. **(E,F)** Mean value (± SEM) with individual data showing the normalized amplitude of N2 **(E)** and the N2/N1 ratio **(F)** during each treatment. *p < 0.05 versus control (ACSF); *n* = 6 in each group.

Administration of the α2-AR antagonist, yohimbine (Yohim, 100 μM), had no effect on facial stimulation-evoked MF-GC synaptic transmission (Supplementary Figure 1). However, additional application of 100 μM Yohim completely revered the NA-induced decrease in amplitude and AUC of N1 (Figures 5A,B). In the presence of a mixture of Yohim and NA, the normalized amplitude of N1 increased from 78.5 ± 5.1% (NA, 25 μM) to 99.3 ± 5.4% [NA + Yohim; *F* (2, 28) = 10.64, *P* = 0.042; *n* = 6] of baseline [ACSF: 101.1 ± 3.8%; *F* (1, 7) = 0.37, *P* = 0.66; *n* = 6; Figure 5C], and the normalized AUC of N1 increased from 79.3 ± 5.1% (NA, 25 μM) to 101.2 ± 5.0% [NA + Yohim; *F* (2, 39) = 11.75, *P* = 0.023; *n* = 6] of baseline [ACSF: 100.2 ± 4.0%; *F* (1, 5) = 0.04, *P* = 0.86; *n* = 6; Figure 5D]. Additional perfusion of Yohim also reversed the NA-induced inhibition of N2. The normalized amplitude of N2 increased from 89.8 ± 5.1% (NA, 25 μM) to 100.9 ± 5.5% [NA + Yohim; *F* (2, 19) = 9.42, *P* = 0.035; *n* = 6] of baseline [ACSF: 101.3 ± 4.0%; *F* (1, 5) = 0.05, *P* = 0.82; *n* = 6; Figure 5E], and the N2/N1 ratio decreased from 114.4 ± 5.4% (NA, 25 μM) to 101.4 ± 5.3% [NA + Yohim; *F* (3, 54) = 15.45, *P* = 0.017; *n* = 6] of baseline [ACSF: 99.9 ± 4.3%; *F* (1, 7) = 1.07, *P* = 0.37 versus ACSF; *n* = 6; Figure 5F].

**FIGURE 6:**
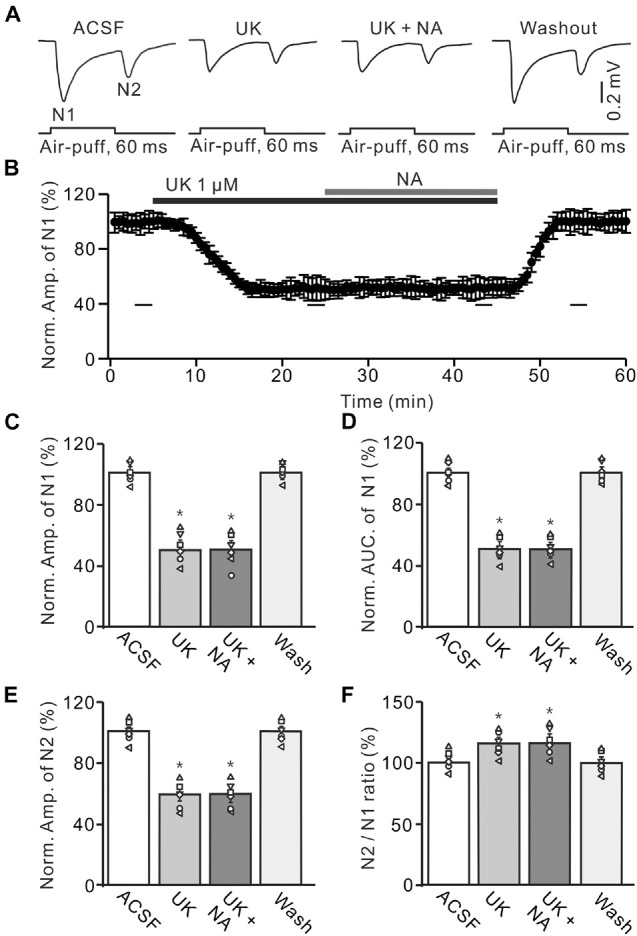
Effect of α2-AR agonist, UK14304 (UK) on the NA induces inhibition of the MF-GC synaptic transmission. **(A)** Representative field potential traces showing the facial stimulation (60 ms, 60 psi) evoked responses in a mouse cerebellar GL during treatment with ACSF, UK14304 (UK; 1 μM), UK + NA (25 μM), and recovery (washout). **(B)** Summary of data showing the time of course of normalized amplitude of N1 during treatment with ACSF, UK14304, UK + NA and recovery (washout). **(C,D)** Mean value (± SEM) with individual data showing the normalized amplitude **(C)** and AUC **(D)** of N1 during each treatment. **(E,F)** Mean value (± SEM) with individual data showing the normalized amplitude of N2 **(E)** and the N2/N1 ratio **(F)** during each treatment. *p < 0.05 versus control (ACSF). *n* = 6 in each group.

We further examined the effect of a highly selective α2-AR agonist, UK14304, on the facial stimulation-evoked MF-GC synaptic transmission to observe whether pharmacological activation of α2-ARs could induce depression of MF-GC synaptic transmission. In the presence of UK14304 (1 μM), the amplitude of N1 decreased to 51.4 ± 5.8% of baseline [ACSF: 99.8 ± 4.0%; *F* (2, 28) = 10.74, *P* = 0.022; *n* = 6], and the AUC of N1 decreased to 52.6 ± 4.9% of baseline [ACSF: 100.1 ± 4.1%; *F* (2, 19) = 8.99, *P* = 0.014; *n* = 6]. Notably, additional application of NA failed to induce further inhibition of MF-GC synaptic transmission (Figures 6A,B). In the presence of a mixture of UK14304 and NA, the amplitude of N1 was 51.8 ± 6.1% [UK14304 + NA; *F* (1, 15) = 6.38, *P* = 0.045; *n* = 6] of baseline (ACSF: 99.8 ± 4.0%; *n* = 6), which was similar to that observed in the presence of UK14304 alone [UK14304: 51.4 ± 5.8%; *F* (1, 7) = 0.41, *P* = 0.72; *n* = 6; Figure 6C], and the normalized AUC of N1 was 52.3 ± 4.4% [UK14304 + NA; *F* (3, 63) = 16.84, *P* = 0.012; *n* = 6] of baseline (ACSF: 100.1 ± 4.1%; *n* = 6), which was not significantly different from that observed in the presence of UK14304 alone [UK14304: 52.3 ± 4.9%; *F* (1, 5) = 0.14, *P* = 0.23; *n* = 6; Figure 6D]. In the presence of UK14304 and NA, the normalized amplitude of N2 was 61.8 ± 5.0% [UK14304 + NA; *F* (2, 28) = 12.76, *P* = 0.033; *n* = 6] of baseline (ACSF: 100.2 ± 4.5%; *n* = 6), which was not significantly different from that observed in the presence of UK14304 alone [UK14304: 60.9 ± 5.1%; *F* (1, 7) = 0.32, *P* = 0.53; *n* = 6; Figure 6E]. The N2/N1 ratio also increased to 119.4 ± 5.3% of baseline [UK14304 + NA; ACSF: 99.9 ± 4.2%; *F* (3, 54) = 16.53, *P* = 0.011; *n* = 6], which was similar to that observed in the presence of UK14304 alone [UK14304: 118.6 ± 4.7%; *F* (1, 4) = 0.046, *P* = 0.081; *n* = 6; Figure 6F]. The results indicate that activation of α2-AR suppresses the evoked MF-GC synaptic transmission and overwhelms the NA-induced inhibition of MF-GC synaptic transmission.

## Discussion

In this study, we showed that cerebellar surface perfusion of NA induced a concentration-dependent depression of facial stimulation-evoked MF-GC synaptic transmission, which was reversed by additional application of an α-AR antagonist but not reversed by a β-AR antagonist. Furthermore, the NA-induced inhibition of facial stimulation-evoked MF-GC synaptic transmission was reversed by additional application of an α2-AR antagonist but not by an α1-AR antagonist. Moreover, pharmacological activation of α2-AR significantly inhibited the facial stimulation-evoked MF-GC synaptic response and overwhelmed the NA-induced depression.

In the cerebellar cortex, GCs receive excitatory inputs from MFs and inhibitory inputs from Golgi cells (Shambes et al., 1978; Bower and Woolston, 1983; Chadderton et al., 2004). For evaluating the sensory information transmitted by MF-GC synaptic transmission, we here studied the facial stimulation-evoked field potential response in the mouse cerebellar GL in the absence of GABAergic inhibitory inputs (Ma et al., 2019). Consistent with previous studies (Wu et al., 2014; Bing et al., 2015; Ma et al., 2019), air-puff stimulation of the ipsilateral whisker pad induced MF-GC synaptic transmission, which expressed stimulus onset and stimulus offset responses in the absence of GABAergic inhibition. These results indicate that tactile mechanoreceptors generate the receptor potentials at both stimulus onset and offset, which suggests that the sensory stimulation-evoked MF-GC synaptic transmission is high-fidelity and reliably reflects the encoded sensory information (Arenz et al., 2008; van Beugen et al., 2013; Bing et al., 2015).

Previous studies showed that NAergic afferents originate in the LC and distribute throughout the cerebellar cortical GL, PC, and molecular layers (Kimoto et al., 1978; Schroeter et al., 2000). Morphological studies have shown that both α-ARs and β-ARs are present in the cerebellar cortex (McCune et al., 1993). We previously found that NA regulates spontaneously complex spikes activity of cerebellar PCs via activation of α2-ARs *in vivo* in mice (Sun et al., 2019). Our results in this study show that cerebellar surface perfusion of NA produces a concentration-dependent inhibition of synaptic transmission convey sensory information in the cerebellar GL. The NA-induced depression of the evoked MF-GC synaptic transmission was reversed by additional application of an α2-AR antagonist and was mimicked by activation of α2-ARs. These results indicate that NA activates α2-ARs, which results in a depression of the facial stimulation-evoked MF-GC synaptic transmission in the mouse cerebellar cortex. In addition, our results show that blockade of α2-AR has less effect on the sensory stimulation-evoked MF-GC synaptic transmission, suggesting that there is less α2-AR activation under these experimental conditions.

α2-Adrenoceptors are coupled to a wide variety of second messenger systems via G_i/o_-proteins, which negatively regulate the activity of adenylyl cyclases and inhibit voltage-gated Ca^2+^ channel activity (Limbird, 1988). Activation of α2-ARs suppresses the production of cAMP-dependent protein kinase activity, leading to the activation of protein phosphatase 1, which plays an inhibitory role in synaptic transmission through modifying α-amino-3-hydroxy-5-methyl-4-isoxazole-propionica (AMPA) receptors (Mulkey et al., 1994; Yan et al., 1999). Activation of α2-ARs reduces the phosphorylation of AMPA receptors via the PKA signaling pathway, resulting in the inhibition of synaptic transmission (Yi et al., 2013). In the cerebellar cortex, α2-ARs play critical roles in information processing and motor coordination skills (Lähdesmäki et al., 2002). A previous study demonstrated that activation of α2-ARs suppresses presynaptic glutamate release from mitral cells by a G_i/o_-protein-mediated inhibition of Ca^2+^ channels in the mouse olfactory bulb (Huang et al., 2018). We previously found that NA inhibits complex spike activity via a presynaptic PKA signaling pathway *in vitro* (Cui et al., 2020). Our results here demonstrate that NA depresses the amplitude of N1 and N2, which is accompanied by an increase in the N2/N1 ratio, suggesting that NA modulates the facial stimulation-evoked glutamate release at the MF-GC synapse. Since the N2/N1 ratio is inversely correlated with the probability of vesicular release, we proposed that the NA-induced depression of MF-GC synaptic transmission by reducing presynaptic glutamate release from mossy fiber terminals (Mennerick and Zorumski, 1995; Hashimoto and Kano, 1998). In addition, we studied the effect of NA on the facial stimulation-evoked MF-GC synaptic transmission in urethane anesthetized mice. We could not exclude the possible effect of urethane on the sensory-evoked MF-GC synaptic transmission. However, administration of urethane produces inhibition of neuronal excitability by activation of the barium-sensitive potassium leak conductance, without effects on excitatory glutamate mediated synaptic transmission (Sceniak and Maciver, 2006; Chu et al., 2011). Therefore, urethane anesthesia might produce less effect on the facial stimulation-evoked MF-GC synaptic transmission *in vivo* in mice.

Cellular mechanisms of motor learning in the cerebellum are long-term depression (LTD) and potentiation (LTP) at PF–PC, MF-GC, and MLI–PC synapses (Ito and Kano, 1982; Ito, 1989; Roggeri et al., 2008; Bing et al., 2015). It has been shown that tactile stimulation of the whisker pad induces long-term synaptic plasticity in MF-GC synapses in anesthetized rats, which suggests that MF-GC synaptic transmission and plasticity are critical for sensory information-dependent motor learning in rodents (Roggeri et al., 2008). Importantly, NAergic inputs to the cerebellum have been implicated in cerebellum-dependent motor learning (McCormick and Thompson, 1982; Keller and Smith, 1983; Watson and McElligott, 1984; Pompeiano, 1998). Our present results show that NA significantly depresses sensory stimulation-evoked MF-GC synaptic transmission, which suggests that cerebellar NAergic inputs modulate synaptic transmission conveying sensory information through MF–GC synapses. In addition, NAergic inputs have been found play critical roles in sensory signal processing, as well as the facilitation of motor coordination and motor learning function (McCormick and Thompson, 1982; Keller and Smith, 1983; Watson and McElligott, 1984; Pompeiano, 1998; Waterhouse and Navarra, 2019). Thus, the NA-induced depression of MF-GC synaptic transmission may directly contribute to sensory information-dependent motor tasks. Since GCs transmit sensory information to PCs through PFs (Ito and Kano, 1982), the NA-induced depression of MF-GC synaptic transmission may modulate MF-PC synaptic plasticity and motor learning by down regulating PF excitatory inputs onto PCs. While further experiments are required to further understand the effects of NAergic inputs on cerebellar physiology, our results provide important insights into the cellular and synaptic mechanisms of how NA modulates sensory information processing in the cerebellar cortex.

## Data Availability Statement

The raw data supporting the conclusions of this article will be made available by the authors, without undue reservation.

## Ethics Statement

All the experimental procedures were reviewed and approved by the Animal Care and Use Committee of Yanbian University and performed in accordance with the animal welfare guide lines of the National Institutes of Health.

## Author Contributions

D-LQ, HJ, and C-PC designed research. B-XL and G-JZ performed *in vivo* electrophysiological experiments and analyzed data. B-XL and L-NC prepared figures and drafts. D-LQ and C-PC wrote the manuscript. All authors contributed to the article and approved the submitted version.

## Conflict of Interest

The authors declare that the research was conducted in the absence of any commercial or financial relationships that could be construed as a potential conflict of interest.

## Publisher’s Note

All claims expressed in this article are solely those of the authors and do not necessarily represent those of their affiliated organizations, or those of the publisher, the editors and the reviewers. Any product that may be evaluated in this article, or claim that may be made by its manufacturer, is not guaranteed or endorsed by the publisher.

## Abbreviations

NA: noradrenaline
CF: climbing fiber
GABA: γ-aminobutyrate
GC: granule cell
MF: mossy fiber
GL: granular layer
PC: Purkinje cell
AR: adrenoceptor
AUC: area under the curve
LC: locus coeruleus
PKA: protein kinase A
ACSF: artificial cerebrospinal fluid.
