# Supplementary material for: Effect of Noradrenaline on the Facial Stimulation-Evoked Mossy Fiber-Granule Cell Synaptic Transmission in Mouse Cerebellar Cortex
Source: Front Neurosci. 2021 Nov 15;15:785995. doi: 10.3389/fnins.2021.785995 (PMC8634677; doi:10.3389/fnins.2021.785995)
Supplement: Supplementary file 1 [file Image_1.pdf]

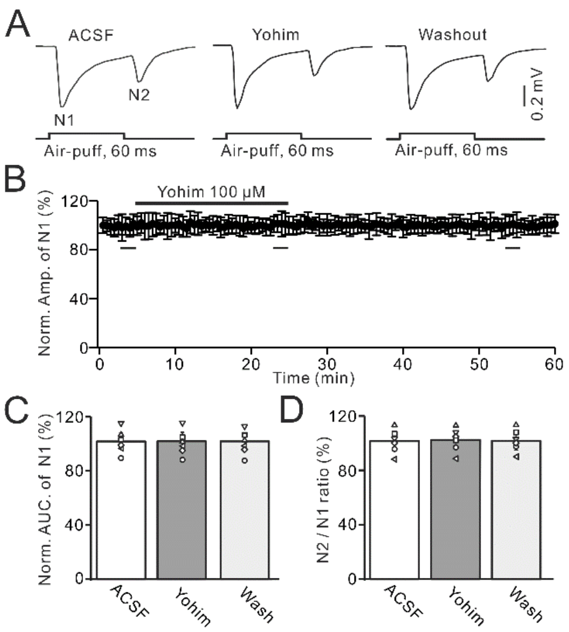

Supplemental Fig. 1. An α2-AR antagonist, yohimbine (Yohim) did not affect the facial stimulation-evoked MF-GC synaptic transmission. (A) Representative field potential traces showing the facial stimulation (60 ms, 60 psi) evoked MF-GC synaptic transmission in a mouse cerebellar GL during treatment with ACSF, yohimbine (Yohim; 100 μM), and recovery (washout). **(B)** Bar graphs with individual data showing the time course of normalized amplitude of N1 during treatment with ACSF, yohimbine (Yohim; 100 μM), and recovery (washout). (**C)** Mean value (± S.E.M) with individual data showing the normalized and AUC of N1 in each treatment. **(D)** Bar graph with individual data showing the normalized N2/N1 ratio (F) in each treatment. * $p < 0.05$ versus control (ACSF); n = 6 in each group.
